# Efatutazone and T0901317 exert synergistically therapeutic effects in acquired gefitinib‐resistant lung adenocarcinoma cells

**DOI:** 10.1002/cam4.1440

**Published:** 2018-03-23

**Authors:** Jie Ni, Lei‐lei Zhou, Li Ding, Xue‐qin Zhang, Xia Zhao, Huizi Li, Haixia Cao, Siwen Liu, Zhuo Wang, Rong Ma, Jianzhong Wu, Jifeng Feng

**Affiliations:** ^1^ Nanjing Medical University Affiliated Cancer Hospital Jiangsu Cancer Hospital Jiangsu Institute of Cancer Research Nanjing Jiangsu 210000 China; ^2^ Department of Oncology Huai'an First People's Hospital Nanjing Medical University Huai'an Jiangsu China; ^3^ The Jiangsu Province Research Institute for Clinical Medicine The First Affiliated Hospital with Nanjing Medical University Nanjing 210029 China; ^4^ Hebei Provincial Children's Hospital Hebei China; ^5^ Department of Oncology First People's Hospital of Yancheng Fourth Affiliated Hospital of Nantong University Yancheng 224001 China

**Keywords:** Efatutazone, gefitinib‐resistance, lung adenocarcinoma, peroxisome proliferator‐activated receptor gamma, T0901317

## Abstract

The development of acquired EGFR‐TKI therapeutic resistance is still a serious clinical problem in the management of lung adenocarcinoma. Peroxisome proliferator activated receptor gamma (PPAR *γ*) agonists may exhibit anti‐tumor activity by transactivating genes which are closely associated with cell proliferation, apoptosis, and differentiation. However, it remains not clear whether efatutazone has similar roles in lung adenocarcinoma cells of gefitinib resistant such as HCC827‐GR and PC9‐GR. It has been demonstrated by us that efatutazone prominently increased the mRNA and protein expression of PPAR *γ*, liver X receptor alpha (LXR *α*),as well as ATP binding cassette subfamily A member 1 (ABCA1). In the presence of GW9662 (a specific antagonist of PPAR *γ*) or GGPP (a specific antagonist of LXR *α*), efatutazone (40 *μ*mol/L) restored the proliferation of both HCC827‐GR and PC9‐GR cells and obviously inhibited the increased protein and mRNA expression of PPAR‐gamma, LXR‐alpha, and ABCA1 induced by efatutazone. LXR *α* knockdown by siRNA (si‐LXR *α*) significantly promoted the HCC827‐GR and PC9‐GR cells proliferation, whereas incubation efatutazone with si‐LXR *α* restored the proliferation ability compared with the control group. In addition, combination of efatutazone and LXR *α* agonist T0901317 showed a synergistic therapeutic effect on lung adenocarcinoma cell proliferation and PPAR gamma, LXR A and ABCA1 protein expression. These results indicate that efatutazone could inhibit the cells proliferation of HCC827‐GR and PC9‐GR through PPAR *γ*/LXR *α*/ABCA1 pathway, and synergistic therapeutic effect is achieved when combined with T0901317.

## Introduction

Lung cancer is the most common malignant tumor in the world, and it is the number one killer of cancer 1. Lung cancer consists of two types: small cell lung cancer and nonsmall cell lung cancer (NSCLC). Lung adenocarcinoma (LAC), which is the most common subtype of NSCLC, accounts for 85% of the cancer 2. Patients with activated‐type epidermal growth factor receptors (EGFRs) mutations have covered a new chapter in molecularly targeted therapies and have been specifically targeted by tyrosine kinase inhibitor (TKI) 3. Gefitinib is recommended for the treatment of patients with EGFR mutations in NSCLC patients. About 80% of advanced NSCLC patients benefit from the clinical application of EGFR TKI, which has led to the revolution in the lung cancer therapy 4. Nevertheless, acquired resistance to TKI is the main reason for the failure of the treatment and most patients do not respond to the first generation of TKI after 10–16 months of treatment 5. In previous articles, PTEN deletion, MET amplification, T790M EGFR 20 exon mutations, and consequent AKT over‐activation may be the potential mechanisms of acquired resistance to gefitinib 6, 7, 8. Therefore, there is an urgent need to develop effective new chemotherapeutic drugs.

Nuclear hormone receptors, such as pregnant X receptors (PXRs), liver X receptors (LXRs), as well as peroxisome proliferator activated receptors (PPARs), induce transcriptional activity through binding with lipophilic hormones such as thyroid hormones and steroids 9. PPAR*γ*, which is a ligand‐activated transcription factor and also known as NR1C3, is a subtype of the PPAR family and the agonists of PPAR*γ* have developed into a new type of anticancer drugs 10. PPAR*γ* may be activated by some synthetic thiazolidinediones (TZD), such as antidiabetic drug pioglitazone, rosiglitazone, and troglitazone.

Similarly, PPAR*γ* may also be activated by fatty acids, eicosanoid derivatives, as well as prostaglandins 11, 12. Several studies have confirmed that troglitazone, the first‐generation PPAR*γ* ligand of thiazolidinedione, has shown significant antiproliferative effects in prostate cancer 13 and cervical cancer 14. In respects of activation of PPAR response–element and inhibition of cancer cell growth, efatutazone, the new third‐generation PPAR*γ* agonist of thiazolidinedione, is 500 times higher effects than that of troglitazone 15. In advanced cancer patients, the results of efatutazone I phase clinical trial show disease control effect and acceptable tolerability 16. Therefore, this preparation shows great potential for cancer treatment.

Another class of nuclear hormone receptors‐LXRs has two subtypes, namely LXRa and LXR*β*. It is reported that LXRs are potential targets for the prevention and treatment of breast cancer, prostate cancer, liver cancer, ovarian cancer, skin cancer, lung cancer, and colorectal cancer, 17, 18. Both the premalignant lesions in the gallbladder in LXRb knockout animals and the elevated proliferation markers expression in colon tissue further confirmed the role of LXRs and their ligands in the initiation and progression of cancers 19, 20. Previous studies have shown that the activation of nuclear receptors PPAR*γ* and LXR*α* is related to the growth inhibition of prostate cancer 9. However, it is not clear whether efatutazone shows similar proliferation inhibition effect in acquired gefitinib resistant lung adenocarcinoma cells. This problem has been studied in this study. Moreover, we explored whether PPAR*γ* agonist efatutazone and the LXR*α* agonist T0901317exert similar synergistic effects on proliferation in lung cancer cells.

## Materials and Methods

### Reagents

Efatutazone was purchased from MedChemExpress (New Jersey). T0901317 was purchased from SigmaeAldrich (St. Louis). Before added to cell cultures, efatutazone and T0901317 were prepared in dimethyl sulfoxide (DMSO) in the vitro analyses.

### Cell culture

HCC827 cells that carry EGFR exon 19 deletion (Del E746‐A750) are lung adenocarcinoma cell lines, which were purchased from the cell bank of the Shanghai Academy of life sciences, Chinese Academy of Sciences. Lung adenocarcinoma cell line PC9 was derived from an untreated Japanese patient with an EGFR 19 exon deletion (Del E746‐A750) 21, which is provided by Dr. Zhou (Guangxi Medical University, China). These cells were kept in the environment of 5% CO_2_ at 37°C in RPMI‐1640 medium supplemented with 10% FBS.

### Establishment of the gefitinib‐resistant HCC827‐GR and PC9‐GRsubline cells from HCC827 and PC9 cells

According to previously reported method, HCC827 and PC9 cells were exposed to increasing the concentration of gefitinib in order to establish gefitinib‐resistant subline cells 22. Ultimately, HCC827 and PC‐9 cells produced stable gefitinib resistance: isolated HCC827‐GR and PC9‐GR cell lines were confirmed to resistant to gefitinib independently. These gefitinib‐resistant cell lines were passed more than 25 times with gefitinib, and the resistance was verified by Cell counting kit‐8(CCK‐8) (Dojindo, Japan).

### Analysis of publicly available datasets

The Oncomine ( https://www.oncomine.org) database 23 was used to determine the gene expression of PPARG in lung adenocarcinoma. We used the Oncomine to query PPARG gene and filter the results by selecting lung adenocarcinoma and cancer vs. normal analysis. Three publically available GEO (Gene Expression Omnibus) datasets ( http://www.ncbi.nlm.nih.gov/geo/) GSE74575, GSE38302, GSE59239 and GSE83666 were used to analyze PPARG expression with respect to lung adenocarcinoma EGFR‐TKI‐resistance. The Kaplan–Meier plotter ( http://kmplot.com/analysis/) database was used to assess the effect of 54,675 genes on survival using 2437 lung cancer samples on the HGU133 Plus 2.0 array, Which was used to analyze the correlation between PPARG expression and overall survival (OS) in lung adenocarcinoma 24. The log‐rank *P*‐value and hazard ratio with 95% confidence intervals were also computed.

To analyze the relationship between PPARG mRNA and LXR*α* as well as ABCA1 mRNA levels in lung adenocarcinomas, we acquired and analyzed the data from TCGA dataset using a tool in http://www.cbioportal.org. Specifically, select “Query” on the home page of the website http://www.cbioportal.org, selects “Lung Adenocarcinoma (TCGA, Provisional)” from Select CancerStudy. In the “Select Genomic Profiles,” select “mRNA Expression z‐Score (RNA Seq V2RSEM)” and “protein/phosphoprotein level (RPPA).” In “Enter Gene set,” input “PPARG: EXP < 0,” then click “Submit.” On the next page, click “Protein Change” tab, then change Antibody Type to “mRNA Expression”, click LXR*α* and ABCA1, the corresponding figure will show.

To analyze the relationship between PPARG and LXR*α*, or ABCA1 mRNA levels in lung adenocarcinomas, we obtained the data from TCGA, Provisional using http://www.cbioportal.org. Specifically, on the home page of the website, select “download data,” then, select “LungAdenocarcinoma (TCGA, Provisional),” click “mRNA expression Z‐score (microarray)” from Select Genomic Profiles, and enter gene set “PPARG, LXR*α*, or ABCA1,” select “Transposedata matrix.” Click “Submit,” the PPARG, LXR*α*, and ABCA1 mRNA Z‐scores of 522 cases will appear. The correlation between PPARG and other genes Z‐scores was then analyzed by Pearson's correlation and plotted using GraphPad Prism 7.

### Measurement of cell viability

The cells were plated in 96‐well plates in various gefitinib concentrations with or without different concentrations of efatutazone after 24‐h incubation, after additional 48 h, each wells were determined by CCK‐8 reagents. The absorbance was measured at 450 nm with an ELISA plate reader. The IC_50_ (50% inhibitory concentrations) values 10, 25 were determined according to the percentages.

### Sequencing the EGFR gene

We used the ABI 3500 sequencer (ABI, Massachusetts) to determine the EGFR sequence of HCC827‐GR and PC9‐GR cell, and primer sequences are displayed in Table 1.

**Table 1:** Primer sequence

Primer name	Primer sequence 5′ to 3′
EGFR18‐F	AGCATGGTGAGGGCTGAGGTGAC
EGFR18‐R	ATATACAGCTTGCAAGGACTCTGG
EGFR19‐F	CCAGATCACTGGGCAGCATGTGGCACC
EGFR19‐R	AGCAGGGTCTAGAGCAGAGCAGCTGCC
EGFR20‐F	GATCGCATTCATGCGTCTTCACC
EGFR20‐R	TTGCTATCCCAGGAGCGCAGACC
EGFR21‐F	TCAGAGCCTGGCATGAACATGACCCTG
EGFR21‐R	GGTCCCTGGTGTCAGGAAAATGCTGG
GAPDH‐F	GGAGCGAGATCCCTCCAAAAT
GAPDH‐R	GGCTGTTGTCATACTTCTCATGG
PPARG‐F	GGGATCAGCTCCGTGGATCT
PPARG‐R	TGCACTTTGGTACTCTTGAAGTT
LXR*α*‐F	ACACCTACATGCGTCGCAAG
LXR*α*‐R	GACGAGCTTCTCGATCATGCC
ABCA1‐F	ACAACCAAACCTCACACTACTG
ABCA1‐R	ATAGATCCCATTACAGACAGCG

### Quantitative real‐time PCR (qRT‐PCR)

The quantitative PCR was fulfilled using SYBR Green mix (Life Technologies, Massachusetts). GAPDH was used as an internal control to normalize the amount of total RNA in each sample, and primer sequences are displayed in Table 1. The relative expression was calculated and normalized using the 2^−ΔΔCt^ method 26.

### Western blotting analysis

HCC827‐GR and PC‐9GR cells were lysed with radio immunoprecipitation assay (RIPA) buffer (Invitrogen, Massachusetts) having proteinase inhibitors. After separation in the SDS–PAGE gel, the protein was transferred on a polyvinylidene fluoride (PVDF) membrane. Membranes were blocked in 5% BSA in TBS‐T for 1.5 h and then incubated overnight (4°C) with antibodies against *β*‐Actin (Cell Signaling Technology, Massachusetts), PPAR*γ*, LXR*α*, and ABCA1 (Invitrogen, Massachusetts). After being washed in TBS‐T, membranes were hatched with goat anti‐rabbit HRP‐conjugated secondary antibody for 2 h at room temperature. The blots were visualized by ECL detection (Invitrogen, Massachusetts).

### Colony formation assay

HCC827‐GR and PC‐9GR cell were seeded at a density of 400 cells per well in flat‐bottomed 6‐well plates. After 24 h of incubation, cells were treated with efatutazone (40 *μ*mol/L) alone diluted with the medium to appropriate concentrations. After 14 days, cells were fixed with 4% paraformaldehyde and had 0.1% crystal violet. Visible colonies 27, 28 were counted.

### siRNA transfection

siRNA (Small‐interference RNA) duplexes for LXR*α* 29 were designed and synthesized by RiboBio Co., Ltd. (Guangzhou, China). HCC827‐GR and PC9‐GR cell were transfected with siRNA or negative control using Lipofectamine 2000 (Invitrogen, Massachusetts).

### Statistical analyses

Comparisons between treatment groups were made using two‐tailed unpaired or paired Student's *t*‐tests. Values are presented as means ± SD and analyzed using one‐way analysis of variance, followed by least significant difference (LSD) test for comparisons of group means. The log‐rank test was used for survival analysis, and the Kaplan–Meier method was used to assess survival time distribution. Statistical significance was defined as a *P* value <0.05. The synergy of data is calculated as [(efatutazone + T0901317)−control] ÷ [(efatutazone−control) + (T0901317−control)]. According to this formula, a value less than 0.5 is antagonistic, a value greater than 1.0 is synergistic, while a value of 0.5–1.0 is additive 10.

## Results

### PPARG expressed in gefitinib‐resistant lung cancer cells and lung adenocarcinoma

**Figure 1:**
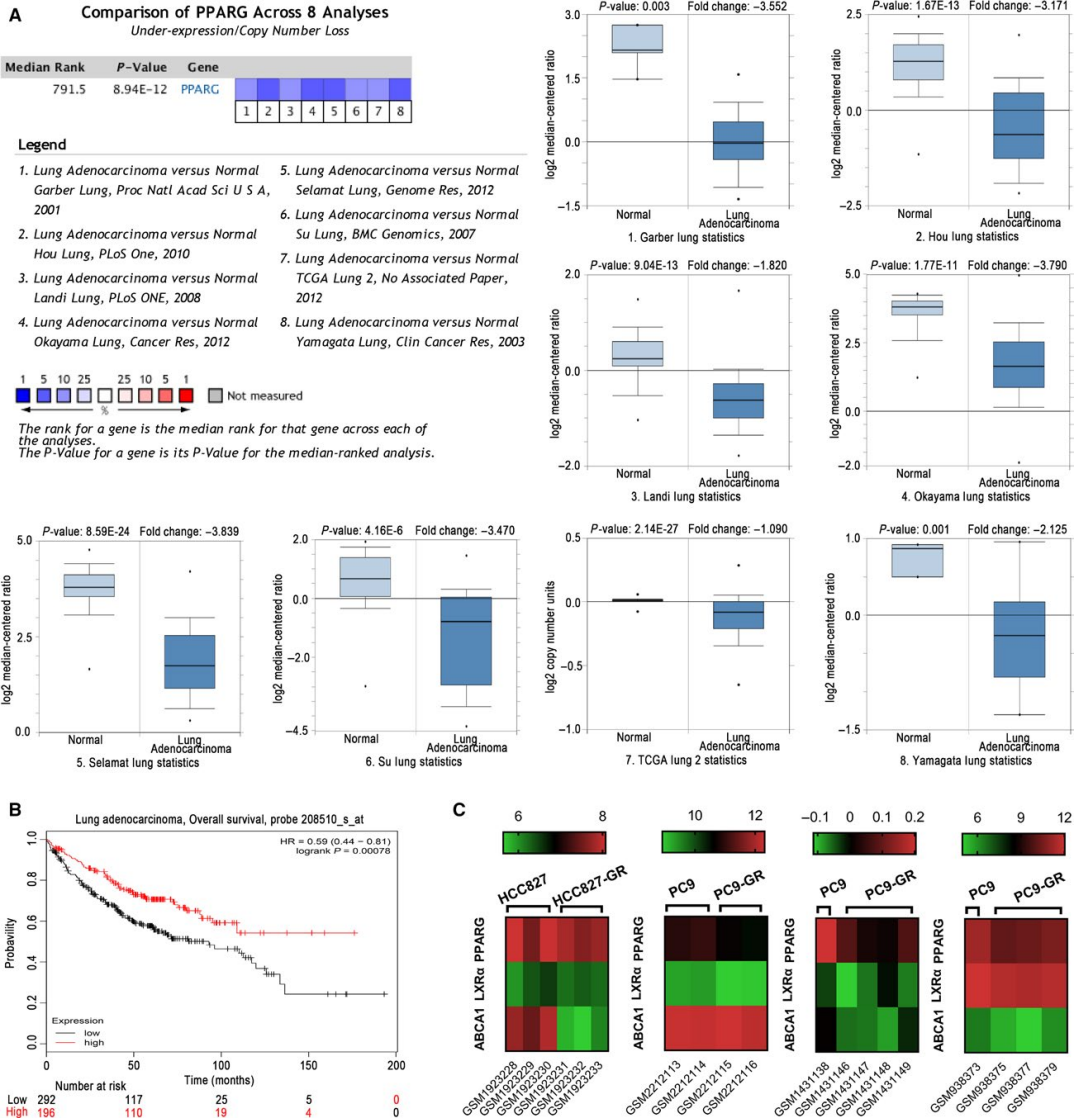
PPARG is down‐regulated in lung adenocarcinoma.(A) Eight analyses were evaluated in comparison with the mRNA expression of PPARG between lung adenocarcinoma and normal tissue. Values above the average were considered PPARG lower expression (blue). Comparison of the expression of PPARG between lung adenocarcinoma and normal samples in the Garber Lung, Hou Lung, Landi Lung, Okayama Lung, Selamat Lung, Su Lung, TCGA Lung 2, and Yamagata Lung database using Oncomine. (B) The survival curve comparing the patient with high (red) and low (black) PPARG genes expression in lung adenocarcinoma was plotted from Kaplan–Meier plotter database. (C) PPARG, LXR *α*, and ABCA1 genes relative expression value in GSE74575, GSE38302, GSE59239, and GSE83666.

To investigate the expression of PPARG gene in lung adenocarcinoma, we employed the Oncominedatabase ( http://www.oncomine.org) to compare the different levels of PPARG mRNA between cancer and normal tissues (Fig. 1A). These eight representative datasets revealed that PPARG gene expression levels were decreased in lung adenocarcinoma (Fig. 1A). To identify the survival time of patients with lung adenocarcinoma, we applied the Kaplan–Meier plotter and log rank analysis. The results showed that PPARG was positively associated with survival time in lung adenocarcinoma (HR = 0.59, *P* = 0.00078) (Fig. 1B). Then, we investigate levels of PPARG, LXR*α*, and ABCA1 in gefitinib‐resistant lung cancer cells in datasets downloaded from GEO database ( http://www.ncbi.nlm.nih.gov/geo/). The results showed that PPARG, LXR*α*, and ABCA1 down‐regulated in gefitinib‐resistant LAC cells: HCC827‐GR and PC9‐GR cells (Fig. 1C).

We therefore hypothesized that PPARG, LXR*α*, and ABCA1 were related to tumor‐suppressive effects against acquired gefitinib‐resistant lung adenocarcinoma cells, and efatutazone, the novel third‐generation PPARG agonist, could be a potentially useful choice for patients with lung adenocarcinoma.

### Establishment of the gefitinib‐resistant HCC827‐GR and PC9‐GR subline cells from HCC827 and PC‐9 cells

**Figure 2:**
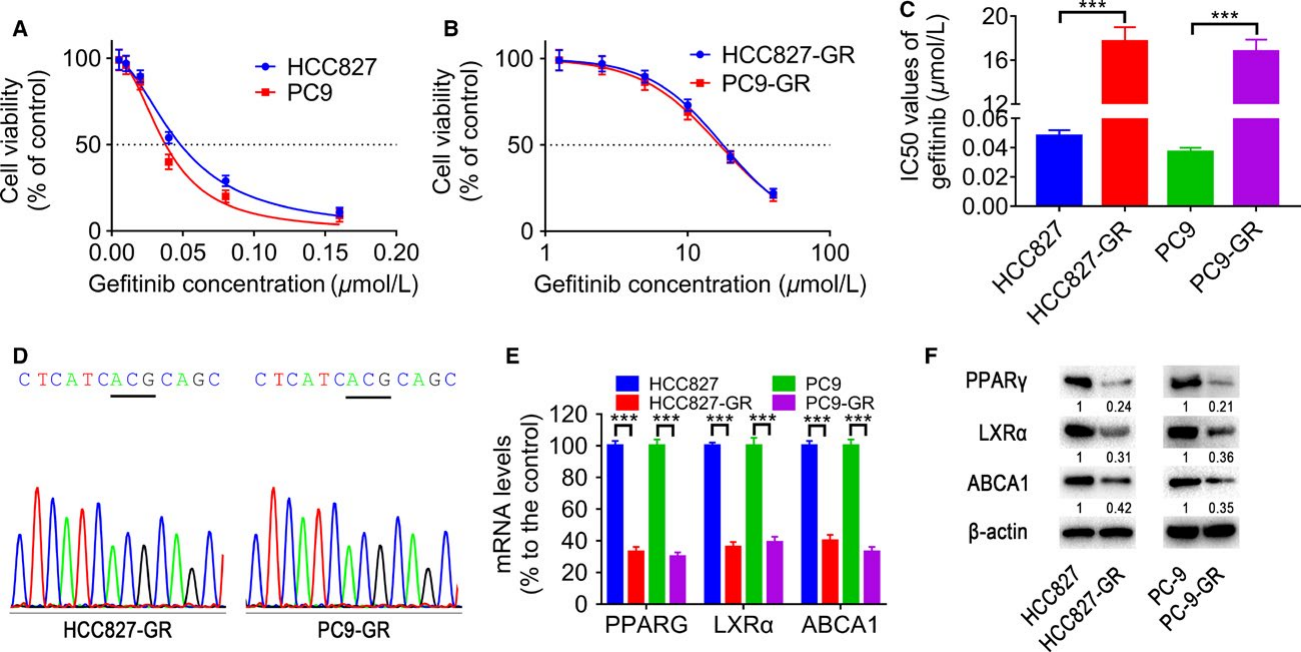
The characterization of HCC827‐GR and PC9‐GR cells. (A) The cytotoxicity of gefitinib (0~0.16 *μ*mol/L) in HCC827 and PC9 cells was determined by CCK‐8 assay. Each point shows the mean ± SD of three independent experiments, performed in triplicate. (B) The cytotoxicity of gefitinib (0~40 *μ*mol/L) in HCC827‐GR and PC9‐GR cells was determined by CCK‐8 assay. Each point shows the mean ± SD of three independent experiments, performed in triplicate. (C) The gefitinib IC50 values of HCC827, HCC827‐GR, PC‐9, and PC‐9‐GR cells. (D) The T790M mutation was not found in HCC827‐GR and PC‐9‐GR cells by direct sequencing. (E) PPARG, LXR *α*, and ABCA1 mRNA levels were examined by real‐time RT‐PCR. (F) Western blot analysis for PPAR *γ*, LXR *α*, and ABCA1. *n* = 3, ***P < 0.001.

To establish gefitinib‐resistant sublines from HCC827 and PC9 cells harboring EGFR activating mutations, we cultured the cells in increasing concentrations of gefitinib within 6 months, as described in Results section. We used CCK‐8 assay to confirm that the new cell line, HCC827‐GR and PC9‐GR, did not show growth suppression in response to exposure to gefitinib (Fig. 2A and B). This cell line in the absence of gefitinib medium serially passaged over 15 generations, no changes in sensitivity to gefitinib 6. The IC50 values of gefitinib for HCC827, HCC827‐GR, PC‐9, and PC‐9‐GR cells were 0.048 ± 0.004 *μ*mol/L, 17.70 ± 1.3 *μ*mol/L, 0.037 ± 0.003 *μ*mol/L, 16.78 ± 1.1 *μ*mol/L, respectively (Fig. 2C). Compared with their parental cell lines, the HCC827‐GR and PC9‐GR cell lines were, respectively, 368.8 and 453.5 folds more resistant to gefitinib, implying the sensitivity of the parental cells and the drug resistance of the progeny cells.

Previous studies reported the association between the mutation of EGFR T790M and acquired resistance to gefitinib in patients 21. We performed DNA sequencing to examine genetic alterations of EGFR at exons 18–22, including the well‐known T790M mutation, and mutation of EGFR T790M at exon 20 was not observed in the HCC827‐GR and PC9‐GR cells. The general mechanism of acquired resistance to HCC827‐GR and PC9‐GR cells was ruled out (Fig. 2D).

We compared levels of PPARG, LXR*α*, and ABCA1 mRNA in parental‐sensitive cell lines, and offspring resistant cell lines by qRT‐PCR. The expressions of PPARG, LXR*α*, and ABCA1 decreased in offspring resistant cell lines compared with their parental‐sensitive cells (Fig. 2E). The PPARG, LXR*α*, and ABCA1 protein expression levels were consistent with mRNA expression levels (Fig. 2F). These findings revealed that down‐regulation of PPAR*γ* might be involved in acquired resistance to EGFR‐TKI in lung adenocarcinoma.

### Efatutazone inhibited the proliferation of HCC827‐GR and PC9‐GR cells

**Figure 3:**
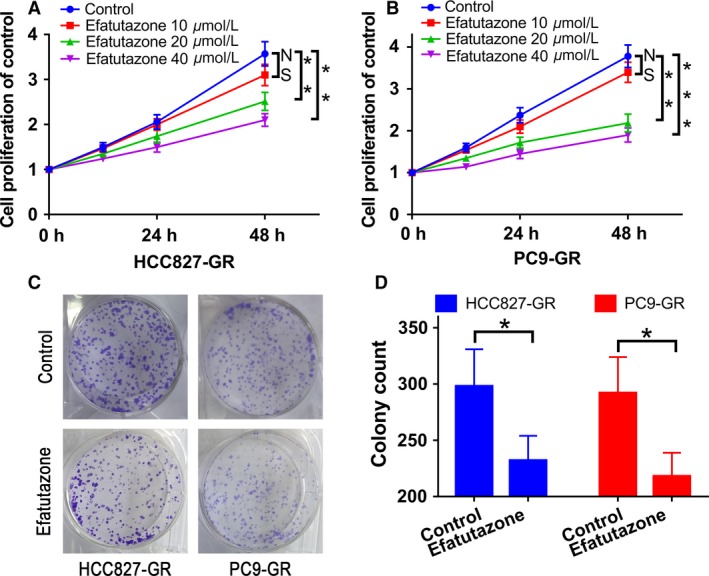
Efatutazone inhibits the cell proliferation in HCC827‐GR and PC9‐GR cells.(A and B) Effect of efatutazone (0–40 *μ*mol/L) on cell proliferation in HCC827‐GR and PC9‐GR cells after incubation for 12, 24, and 48 h. Values are means ± S.D., *n* = 3; means from the same incubation time not sharing a letter differ significantly (*P* < 0.05). (C and D) The colony formation assay of HCC827‐GR and PC9‐GR cells. *n* = 3, *P < 0.05; **P < 0.01; ***P < 0.001.

We explored whether efatutazone could inhibit proliferation of gefitinib‐resistant lung cancer cell lines: HCC827‐GR and PC9‐GR cells. As expected, the proliferation of HCC827‐GR cells was markedly decreased after incubation with efatutazone (0~40 *μ*mol/L) for 12, 24, and 48, and the inhibitory effect of efatutazone increased with increasing concentration (Fig. 3A). Similar results were observed in PC9‐GR cells (Fig. 3B).

Moreover, the colony forming assays demonstrated that efatutazone could inhibit the proliferation of gefitinib‐resistance cells (Fig. 3C and D). These results suggested that efatutazone inhibited the proliferation of HCC827‐GR and PC9‐GR cells in a dose‐dependent manner.

### Efatutazone regulated expressions of PPAR*γ*, LXR*α*, and ABCA1 in HCC827‐GR and PC9‐GR cells

**Figure 4:**
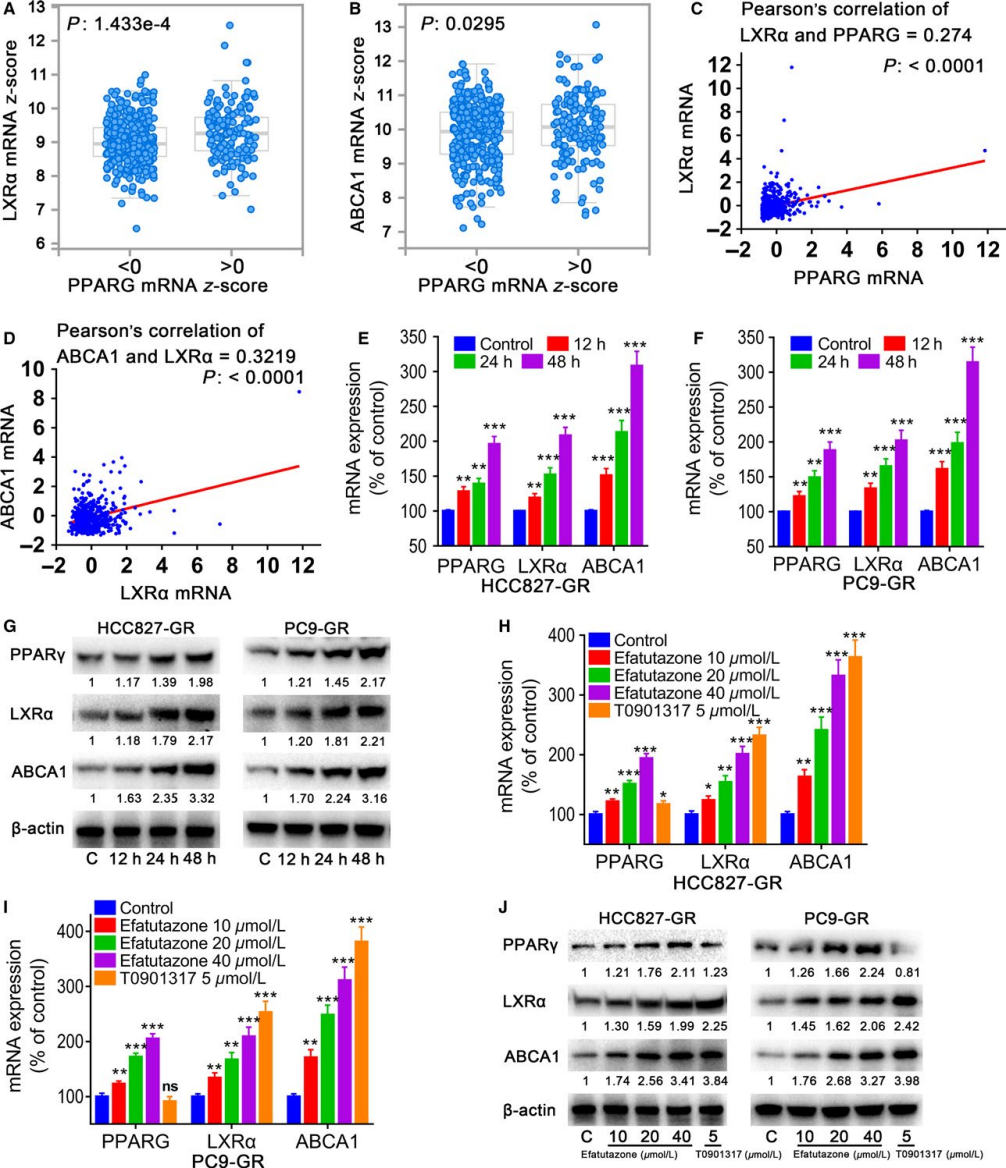
Time course and concentration effects of efatutazone on the expression of PPAR *γ*, LXR *α*, and ABCA1 in HCC827‐GR and PC9‐GR cells.(A) The relationship between PPARG mRNA level and LXR *α* mRNA level in human lung adenocarcinoma. (B) The relationship between PPARG mRNA level and ABCA1 mRNA level in human lung adenocarcinoma. (C) The correlation between PPARG and LXR *α* genes mRNA z‐scores was then analyzed by Pearson's correlation and plotted using GraphPad Prism 7. (D) The correlation between LXR *α* and ABCA1 genes mRNA z‐scores was then analyzed by Pearson's correlation and plotted using GraphPad Prism 7. (E and F) mRNA expression in HCC827‐GR and PC9‐GR cells incubated with 40 *μ*mol/L efatutazone for 12, 24, and 48 h. (G) Protein expression in cells incubated with 40 *μ*mol/L efatutazone for 12, 24, and 48 h. (H and I) mRNA expression in cells incubated with 0–40 mmol/L efatutazone for 48 h. T0901317 (5 *μ*mol/L) serves as positive control. (J) protein expression in cells incubated with 0–40 mmol/L efatutazone for 48 h. T0901317 (5 *μ*mol/L) serves as positive control. *n* = 3, *P < 0.05; **P < 0.01; ***P < 0.001; ns, not significant.

Our data indicated that PPAR*γ* affected cell growth of lung cancer cells expressing LXR*α* and ABCA1, we attempted to examine whether PPAR*γ* regulated the expression of LXR*α* and ABCA1. Analysis for TCGA data by cBioPortal ( http://www.cbioportal.org/public-portal/) elucidated a positive correlation between the expression of PPAR*γ* mRNA and LXR*α* as well as ABCA1 mRNA levels in NSCLC (Fig. 4A and B). To determine whether PPAR*γ* regulates LXR*α* and ABCA1 expression, we first performed cBioPortal to analyze the publically available TCGA data. A significantly positive correlation between PPAR*γ* and LXR*α* as well as ABCA1 mRNA levels was revealed by the Spearman correlation analyses (Fig. 4C and D).

Next, we confirmed this correlation by analyzing qRT‐PCR and Western blot. Based on the results of the proliferation experiment, we chose the concentration of efatutazone with the highest inhibitory (40 *μ*mol/L) to examine effect of efatutazone on expressions of PPAR*γ*, LXR*α*, and ABCA1 in HCC827‐GR cells for 12, 24, and 48 h. Results demonstrated that efatutazone significantly increased expressions of expression of PPAR*γ*, LXR*α*, and ABCA1 at protein and mRNA levels during 48‐h incubation, with the strongest effect at 48 h (Fig. 4E and G). Similar results were observed in PC9‐GR cells (Fig. 4F and G).

Based on the results of time course, we selected the most effective point in time (48‐h incubation) to explore the effect of efatutazone (0–40 *μ*mol/L) on expressions of PPAR*γ*, LXR*α*, and ABCA1 in HCC827‐GR and PC9‐GR cells. We observed that efatutazone significantly increased expressions of PPAR*γ*, LXR*α*, and ABCA1 at the protein and mRNA levels (Fig. 4H and J). Using LXR agonist T0901317 as a positive control, there was no significant change in the expression of PPAR*γ* in HCC827‐GR cells, but the expression of LXR and ABCA1 was significantly increased (Fig. 4H and J). Similar results were obtained in PC9‐GR cells (Fig. 4I and J).

### Inhibition of PPARG and LXR*α* diminished the synergistic effects

**Figure 5:**
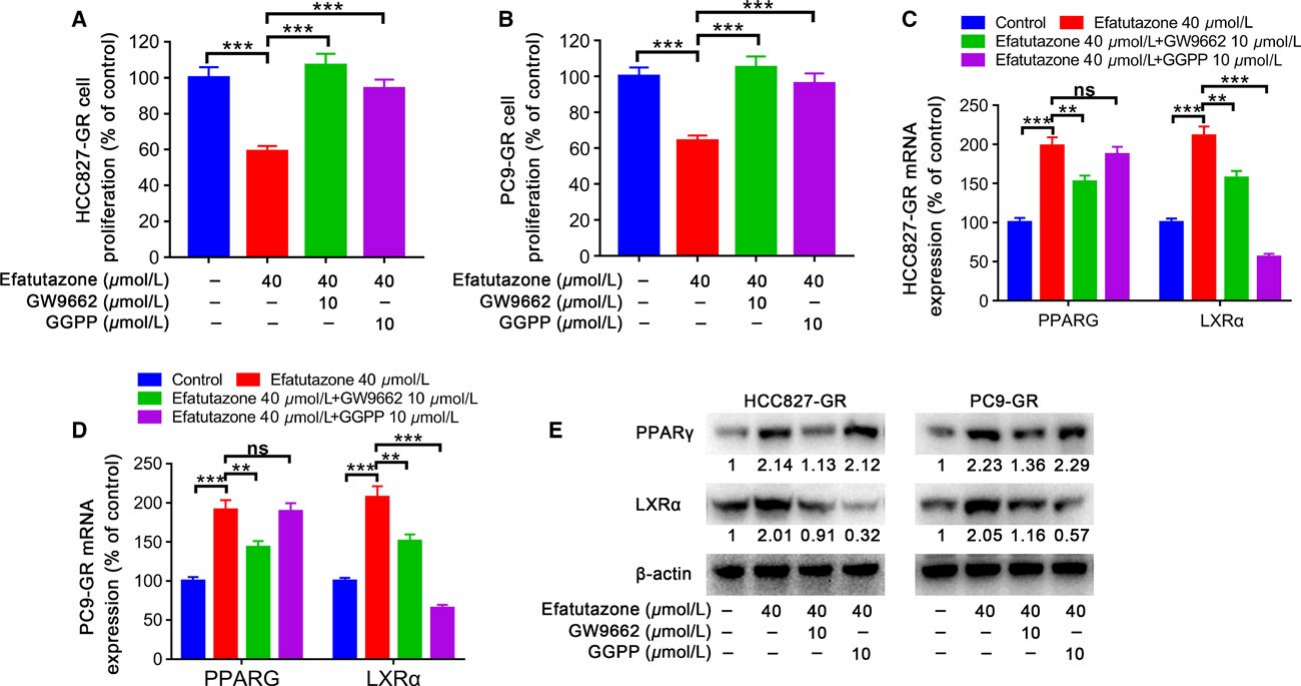
Cell proliferation (A and B), mRNA (C and D), and protein (E) expression of PPAR *γ* and LXR *α* in HCC827‐GR and PC9‐GR cells incubated for 48 h with efatutazone (40 *μ*mol/L) in the presence or absence of an antagonist of PPAR *γ* (GW9662, 10 *μ*mol/L) or LXR *α* (GGPP, 10 *μ*mol/L). *n* = 3, **P < 0.01; ***P < 0.001; ns, not significant.

To confirm synergistic inhibition, we incubated HCC827‐GR cells with efatutazone (40 *μ*mol/L) in the presence or absence of GW9662 10 *μ*mol/L (a selective antagonist of PPAR*γ*) or GGPP 10 *μ*mol/L (a selective antagonist of LXR*α*) for 48 h. As shown in Figure 5A, incubating HCC827‐GR cells with efatutazone and GW9662 or GGPP, the anti‐proliferative effect of efatutazone was effectively reversed and expression of PPAR*γ* and LXR*α* induced by efatutazone was restored (Fig. 5C and E). Similar results were obtained in PC9‐GR cells (Fig. 5B, D, and E).

### Efatutazone inhibited proliferation of HCC827‐GR and PC9‐GR cells via PPAR*γ*‐LXR*α*‐ABCA1 pathway

**Figure 6:**
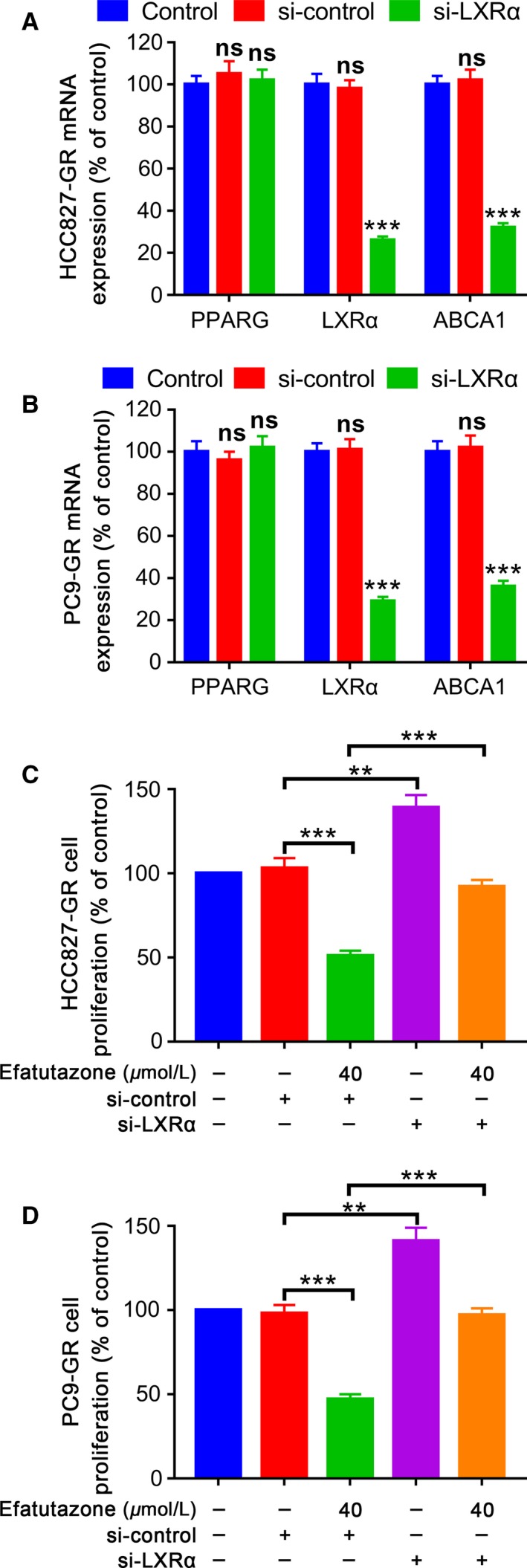
PPAR *γ*, LXR *α*, and ABCA1 mRNA expression levels (A and B) and cell proliferation (C and D) after knockdown of LXR *α*. HCC827‐GR and PC9‐GR cells were transfected with si‐RNA against LXR *α* for 48 h, and mRNA expression was determined using qRT‐PCR. *n* = 3, **P < 0.01; ***P < 0.001; ns, not significant.

We further proved the role of LXR*α* in the antiproliferative effect of efatutazone by the RNA silencing technique. Inhibition expression of LXR*α* in HCC827‐GR and PC9‐GR cells decreased levels of LXR*α* and ABCA1 without change of PPAR*γ* (Fig. 6A and B). To explore the involvement of LXR*α* in the antiproliferative effects of efatutazone in HCC827‐GR and PC9‐GR cells, we transfected with HCC827‐GR and PC9‐GR cells with si‐LXR*α* for 48 h and then treated the two sublines with efatutazone for 48 h. The results suggested that knockdown of LXR*α* significantly increased cell proliferation. In addition, treatment with si‐LXR*α*+efatutazone restored the cell proliferation to control levels (Fig. 6C and D), indicating that LXR*α* might be involved in the antiproliferative effects of efatutazone in HCC827‐GR and PC9‐GR cells.

### Efatutazone and T0901317 synergistically inhibited proliferation of HCC827‐GR and PC9‐GR cells

We then combined efatutazone (40 *μ*mol/L) with T0901317 (5 *μ*mol/L) to evaluate the synergistic effects. Synergistic effects of combined efatutazone (40 *μ*mol/L) with T0901317 (5 *μ*mol/L) on cell proliferation were further analyzed. As illustrated in Table 2, efatutazone inhibited cell proliferation in a concentration‐dependent manner (11.7%, *P* = 0.0189 and 13.3%, *P* = 0.0118, respectively, in HCC827‐GR and PC‐9‐GR cells), and T0901317 significantly inhibited cell proliferation by 8.9% (*P* = 0.0457) and 9.8% (*P* = 0.0331), respectively. The combination of 40 *μ*mol/L efatutazone and 5 *μ*mol/L T0901317 significantly inhibited proliferation of HCC827‐GR and PC9‐GR cells (by 36.2%, *P* = 0.0003 and 38.9%, *P* = 0.0002, respectively), showing the synergistic effects (1.76‐fold and 1.68‐fold of the control, respectively).

**Table 2:** Cell proliferation in HCC827‐GR and PC9‐GR cells incubated with efatutazone in combination with T0901317 for 48 h

Cell	Treatment	Cell viability^a^ (%)	*P* value	Fold of synergy^b^
HCC827‐GR	Control	100	—	—
	Efatutazone 40 *μ*mol/L	88.3 ± 3.5	0.0189	—
	T0901317 5 *μ*mol/L	91.1 ± 3.6	0.0457	—
	Efatutazone 40 *μ*mol/L + T0901317 5 *μ*mol/L	63.8 ± 3.3	0.0003	1.76
PC9‐GR	Control	100	—	—
	Efatutazone 40 *μ*mol/L	86.7 ± 3.4	0.0118	—
	T0901317 5 *μ*mol/L	90.2 ± 3.5	0.0331	—
	Efatutazone 40 *μ*mol/L + T0901317 5 *μ*mol/L	61.1 ± 3.3	0.0002	1.68

a Values are means ± SD, *n* ≥ 3; means without a common letter differ significantly.

b The synergy of data is calculated as [(Efatutazone + T0901317)−control] ÷ [(Efatutazone−control) + (T0901317−control)].

## Discussion

EGFR‐TKIs, such as gefitinib and erlotinib, exert an excellent effect in NSCLCs patients with EGFR mutations. Unfortunately, almost all patients of NSCLCs succumb to relapse due to drug resistance 5. Consequently, searching for novel and effective chemotherapeutic approaches is imminent. It is exciting that using PPAR*γ* agonist efatutazone can not only re‐sensitize the treatment of EGFR‐TKIs, but also may overcome the problem of drug resistance with the increase in dose 10.

It has been shown that LXR*α* and PPAR*γ* heterodimerize with retinoid X receptor (RXR) and activating the two heterodimers (LXR*α*/RXR and PPAR*γ*/RXR) could inhibit the prostate cancer cells proliferation 9.However, it is still not clear whether efatutazone has a similar effect on the gefitinib‐resistant lung adenocarcinoma cells (HCC827‐GR and PC9‐GR). In this article, we confirmed that the antiproliferative activity of efatutazone in HCC827‐GR and PC9‐GR cells may be due to up‐regulating the PPAR*γ*/LXR*α*/ABCA1 pathway. Meanwhile, synergetic effects of efatutazone and T0901317 on lung cancer cells proliferation inhibition and PPAR*γ*/LXR*α*/ABCA1 pathway activation were also proved.

It was indicated by a great deal of evidence that PPAR*γ*‐LXR*α* pathway could mediate the expression of ABCA1 30. In the process of initiation and development of lung adenocarcinoma, the expressions of PPAR, LXR, and ABCA1 were decreasing. On the other hand, the lung adenocarcinoma cells proliferation could be inhibited by the over expression of the three genes above 10, 22.Our findings that efatutazone elevated the expression of PPAR*γ*, LXR*α*, and ABCA1 further proved that efatutazone participates in PPAR*γ*/LXR*α*/ABCA1 pathway in HCC827‐GR and PC9‐GR cells.

Afterward, antagonists and siRNA against LXR*α* were used to affirm that the PPAR*γ*/LXR*α*/ABCA1 pathway participated in inhibition effect by efatutazone on HCC827‐GR and PC9‐GR cells proliferation. It was proved by us that in HCC827‐GR and PC9‐GR cells, the anti‐proliferative effect of efatutazone was reversed by PPAR*γ* antagonist (GW9662) and LXR*α* antagonist (GGPP) and the expression of PPAR*γ*, LXR*α*, and ABCA1induced by efatutazone attenuated. Moreover, in HCC827‐GR and PC9‐GR cells, we also showed that with PPAR*γ* expression stable, si‐LXR*α* depressed LXR*α* and ABCA1 expression levels, which approved that LXR*α* is a downstream target gene of the PPAR*γ*, consistent with previous reports 31. There was another report that the lung adenocarcinoma cells proliferation could be restrained by LXR*α* activation 22. Therefore, cancer cells proliferation may be increased followed by LXR*α* and ABCA1 inactivated. In addition, we demonstrated that the proliferation ability of HCC827‐GR and PC9‐GR cells was increased with si‐LXR*α* treatment, whereas subsequent incubation of HCC827‐GR and PC9‐GR cells with efatutazone reversed si‐LXR*α*‐induced cell proliferation compared with the control groups. The results here obtained using antagonists (GW9662 and GGPP) and by knocking down LXR*α* confirmed that increased expression of LXR*α* was associated with higher sensitivity of lung adenocarcinoma cell proliferation inhibition 29.

Although clinical studies had affirmed the acceptable tolerability of efatutazone, about 51.6% patients still suffered from peripheral edema, which is a widely recognized adverse reaction under efatutazone therapy. In addition, the tumor suppressor effect of efatutazone was also confirmed in advanced cancer patients 16. Besides, in a multicenter phase 1 trial, the combination of Efatutazone and paclitaxel is safe, tolerable, and biologically active 32.

T0901317, a synthetic LXR agonist and a positive control in our research, has been proved to restrained the gefitinib‐resistant lung adenocarcinoma cells growth, and this growth inhibition effect was related to the activated expression of LXR target gene 33. Synergetic proliferation inhibition effect on HCC827‐GR and PC9‐GR cells and increased expression of PPAR*γ*, LXR*α*, and ABCA1 proteins were observed in combination with of the efatutazone (40 *μ*mol/L) and T0901317 (5 *μ*mol/L). This synergistic action further verified that the PPAR*γ*/LXR*α*/ABCA1 pathway played an important role in the anti‐tumor proliferative effect of efatutazone.

In conclusion, our research demonstrated that in lung cancer EGFR‐TKI‐resistant HCC827‐GR and PC9‐GR cells, combination treatment with efatutazone and T0901317 produced a synergistic effect. The PPAR*γ*/LXR*α*/ABCA1 signaling pathway was validated to be involved in the synergistic effect. Our results suggested that the combination of efatutazone with T0901317 could reverse the acquired TKI resistance in HCC827‐GR and PC9‐GR cells, providing a potential therapeutic approach for lung cancer.

## Conflict of Interest

None declared.
